# Progress in Immunization Safety Monitoring — Worldwide, 2010–2019

**DOI:** 10.15585/mmwr.mm7015a2

**Published:** 2021-04-16

**Authors:** Omar Salman, Katherine Topf, Rebecca Chandler, Laura Conklin

**Affiliations:** ^1^Global Immunization Division, Center for Global Health, CDC; ^2^Uppsala Monitoring Centre, WHO Collaborating Centre for International Drug Monitoring, Uppsala, Sweden.

High levels of coverage with safe and effective immunizations are critical to the successful control and prevention of vaccine-preventable diseases worldwide. In addition to stringent standards to regulate the safety of vaccines, robust postlicensure monitoring systems help ensure that the benefits of vaccines continue to outweigh the risks for the populations who receive them. National Expanded Programmes on Immunization (EPI) are typically responsible for identifying and investigating adverse events following immunization (AEFI), including assessment of causality. National regulatory authorities (NRAs) are mandated to perform postlicensure surveillance of adverse drug reactions, including those associated with receipt of vaccines. This report describes global progress toward meeting World Health Organization (WHO) indicators on minimal country capacity for vaccine safety surveillance and coordination of AEFI reporting between countries’ EPI and NRAs. In 2019, among 194 countries, 129 (66.5%) reported having an operational national AEFI causality review committee, compared with 94 (48.5%) in 2010. During 2010–2019, the proportion of countries reporting ≥10 AEFI per 100,000 surviving infants per year (an indicator of country capacity to monitor immunization safety) increased, from 41.2% to 56.2%. In 2019, however, only 46 (23.7%) countries reported AEFI data from both EPI and NRAs. Although global progress has been made toward strengthening systems for vaccine safety monitoring over the past decade, new indicators for monitoring global immunization safety performance are needed to better reflect program functionality. Continued global efforts will be vital to address barriers to routine reporting of AEFI, build national capacity for AEFI investigation and data management, and improve sharing of AEFI data at national, regional, and global levels.

In 2014, WHO’s Global Advisory Committee for Vaccine Safety proposed two performance indicators to assess minimum country capacity for vaccine safety monitoring: 1) having a national causality review committee and 2) reporting ≥10 AEFI per 100,000 surviving infants per year (*1*). WHO monitors annual country progress toward meeting these indicators using aggregate passive AEFI data collected through the collaborative WHO and UNICEF Joint Reporting Form (JRF), a questionnaire for the joint collection of data (*2*). NRAs also report case-based AEFI data to the WHO Collaborating Centre for International Drug Monitoring at the Uppsala Monitoring Centre (UMC) in Sweden through VigiBase, the global database of individual case safety reports (*3*). Coordination of AEFI reporting between EPI and NRAs helps to ensure data quality, completeness, and usability, so that any safety signals* can be detected and responded to quickly (*4*). When country EPI and NRA programs coordinate, the AEFI data reported globally through each system align. To assess the degree of coordination of AEFI reporting between national EPI and NRA programs, publicly available data reported globally during 2010 and 2015–2019 through the JRF were compared with those reported through VigiBase, the pharmacovigilance database developed by UMC (*4*). Reporting to either system is voluntary and varies by year. Countries not reporting to VigiBase or through the JRF during the reporting period were included in the denominator when calculating proportions and considered as not meeting the measured goals. Countries were classified as low, lower middle, upper middle, and high income, based on World Bank income group classifications, to categorize differences in reporting trends (*5*).

In 2010, only 94 (48.5%) of 194 countries reported having an operational national AEFI causality review committee, compared with 126 (64.9%) in 2015, 132 (68.0%) in 2018, and 129 (66.5%) in 2019, representing an increase of 37.2% from 2010 to 2019. In 2019, among 194 WHO member states that reported to the JRF, 167 (86.0%) reported having a national system to monitor AEFI in all age groups. The proportion of countries achieving the indicator of ≥10 AEFI reports per 100,000 surviving infants was higher during 2018 (61.9%) and 2019 (56.2%) than in 2010 (41.2%) in all regions (Table). The largest increase (from seven [14.9%] countries in 2010 to 27 [57.4%] in 2019) occurred in the African Region (AFR). Whereas an increase in the percentage of countries achieving the indicator was reported in all regions from 2010 to 2018 and 2019, declines were observed from 2018 to 2019 in the South-East Asia Region (SEAR) (81.8% to 63.6%), Eastern Mediterranean Region (EMR) (from 57.1% to 52.4%), European Region (EUR) (66.0% to 52.8%), and Western Pacific Region (WPR) (44.4% to 40.7%). The highest percentage of countries achieving the indicator in 2019 was in the Region of the Americas (AMR) (71.4%), followed by SEAR (63.6%); the lowest percentage (40.7%) was in WPR.

**TABLE:** Number of countries reporting ≥10 adverse events following immunization per 100,000 surviving infants, by World Health Organization (WHO) region and year — worldwide, 2010 and 2015–2019*

WHO region	No. of countries in region	No (%),^†^ by year					
		2010	2015	2016	2017	2018	2019
AFR	47	7 (14.9)	13 (27.7)	22 (46.8)	20 (42.6)	27 (57.4)	27 (57.4)
AMR	35	21 (60.0)	22 (62.9)	24 (68.6)	21 (60.0)	25 (71.4)	25 (71.4)
EMR	21	8 (38.1)	9 (42.9)	10 (47.6)	12 (57.1)	12 (57.1)	11 (52.4)
EUR	53	34 (64.2)	34 (43.4)	37 (69.8)	45 (84.9)	35 (66.0)	28 (52.8)
SEAR	11	4 (36.4)	3 (27.0)	7 (63.6)	9 (81.8)	9 (81.8)	7 (63.6)
WPR	27	6 (22.2)	12 (44.4)	11 (40.7)	13 (48.1)	12 (44.4)	11 (40.7)
**All regions**	**194**	**80 (41.2)**	**93 (47.9)**	**111 (57.2)**	**120 (61.9)**	**120 (61.9)**	**109 (56.2)**

**Abbreviations:** AFR = African Region; AMR = Region of the Americas; EMR = Eastern Mediterranean Region; EUR = European Region; SEAR = South-East Asia Region; WPR = Western Pacific Region.

* Data from WHO/UNICEF Joint Reporting Form data for 2010, 2015, 2016, 2017, 2018, and 2019.

^†^ Missing data were included in the denominator.

Among the 194 countries, 164 reported the source of national AEFI data in 2019. The primary data source was EPI for 76 (39.2%) countries, NRA for 25 (12.9%) countries, and both for 46 (23.7%) countries (Figure 1). Seventeen (8.8%) countries^†^ reported that other independent safety monitoring institutions served as the source of the national AEFI data (such as the Vaccine Adverse Event Reporting System in the United States). During 2018, the absolute numbers of AEFI reported through the JRF, compared with those received by VigiBase, varied by country income status (Figure 2) and WHO region (Figure 3). Higher income countries and those in AMR and EUR tended to report more frequently to VigiBase. High-income countries also tended to report more serious AEFI^§^ than did low- and middle-income countries.

**FIGURE 1:**
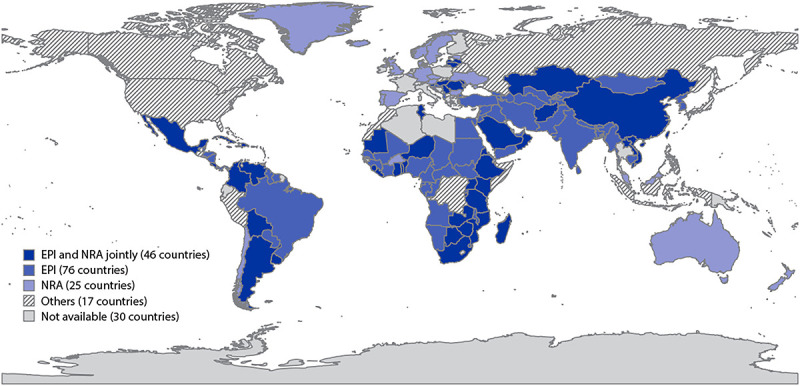
Sources of data for adverse events following immunization reported on the WHO/UNICEF Joint Reporting Form — worldwide, 2019 **Abbreviations:** EPI = Expanded Programmes on Immunization; NRA = national regulatory authorities; WHO = World Health Organization.

**FIGURE 2:**
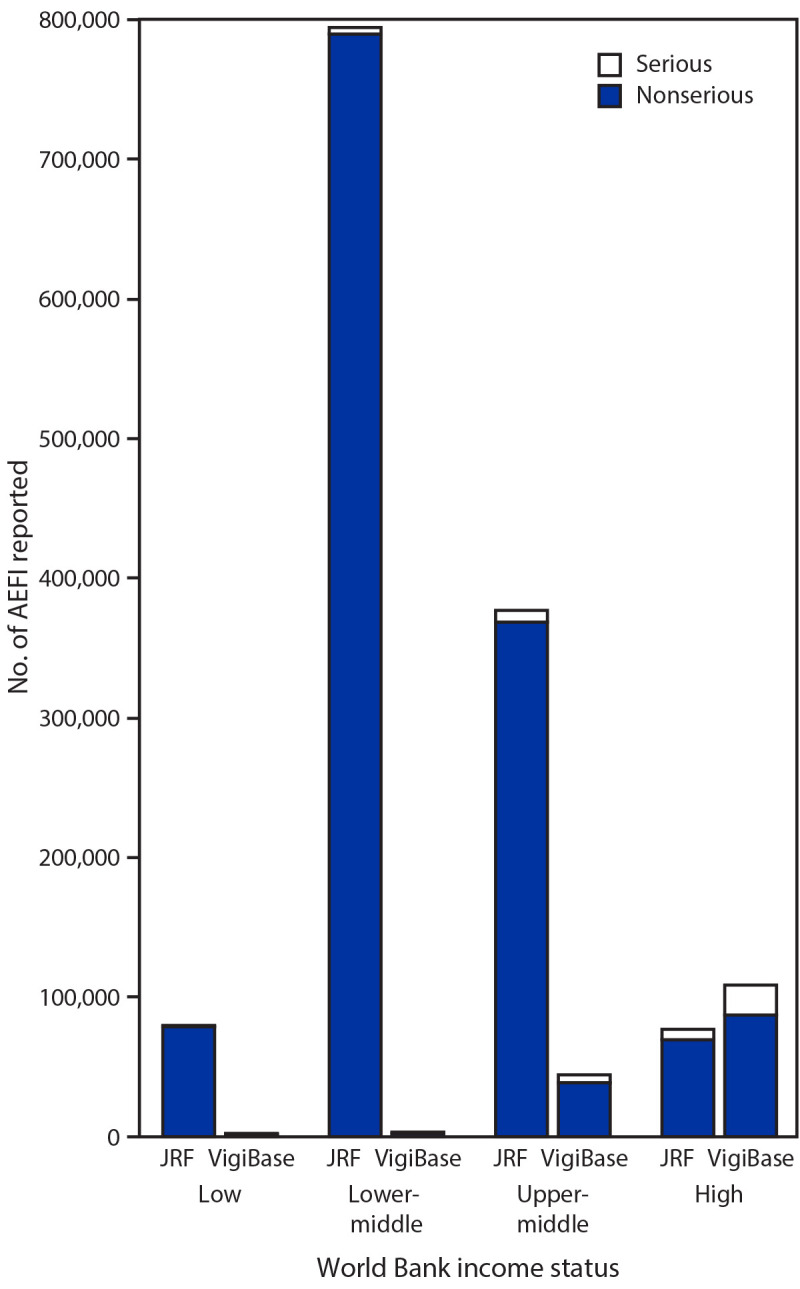
Serious and nonserious* adverse events following immunization (AEFI) reported globally to the WHO/UNICEF Joint Reporting Form (JRF) (164 countries) and VigiBase (95 countries), by country income status^†^ — worldwide, 2018 **Abbreviation:** WHO = World Health Organization. * Serious AEFI are those that are life-threatening, result in hospitalization or a prolongation of hospitalization, result in persistent or substantial disability, or where the outcome is a birth defect or death, as defined by the World Health Organization. A nonserious AEFI does not pose a potential risk to the health of the recipient. † Country income classification based on World Bank Country and Lending Groups classification gross national income (GNI) data (low: GNI ≤$1,035; lower-middle: GNI = $1,036–$4,045; upper-middle: GNI = $4,046–12,535; high: GNI ≥12,536). https://datahelpdesk.worldbank.org/knowledgebase/articles/906519-world-bank-country-and-lending-groups

**Figure 3:**
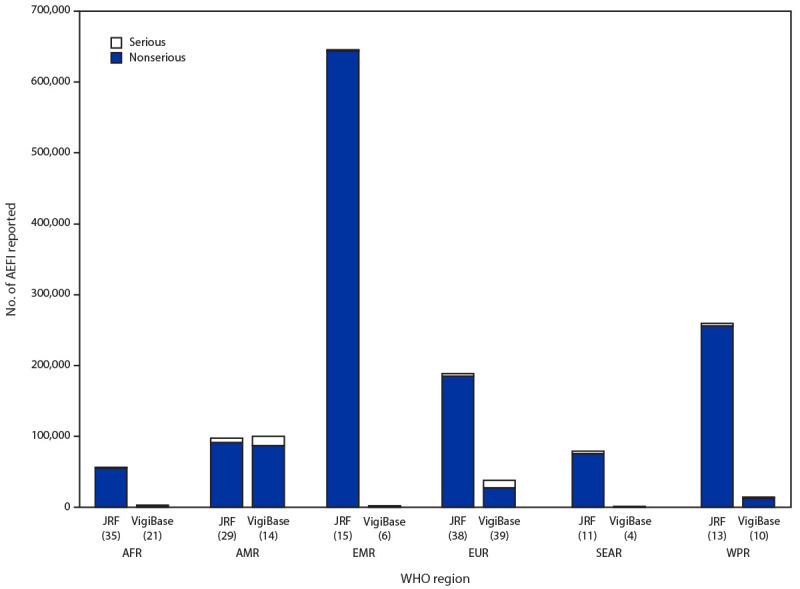
Serious and non-serious* adverse events following immunization (AEFI) reported globally to the WHO/UNICEF Joint Reporting Form (JRF) (164 countries) and VigiBase (95 countries), by WHO region — worldwide, 2018 **Abbreviations:** AFR = African Region; AMR = Region of the Americas; EMR = Eastern Mediterranean Region; EUR = European Region; SEAR = South-East Asia Region; WHO = World Health Organization; WPR = Western Pacific Region. * Serious AEFI are those that are life-threatening, result in hospitalization or a prolongation of hospitalization, result in persistent or substantial disability, or where the outcome is a birth defect or death, as defined by the World Health Organization. A nonserious AEFI does not pose a potential risk to the health of the recipient.

## Discussion

Modern vaccines are safe and effective. However, because vaccines are targeted toward healthy persons seeking to reduce their risk for disease, national immunization programs need to be able to detect and respond to any vaccine safety concerns, ensure effective vaccine safety monitoring is in place, and maintain public confidence in immunization programs. Most WHO regions made progress toward achieving the minimum capacity for vaccine safety monitoring in 2018 and 2019, when compared with 2010, by establishing national AEFI causality committees and reporting ≥10 AEFI per 100,000 surviving infants. Progress has been particularly notable in AFR, where WHO implemented vaccine safety trainings, standardized AEFI data collection tool development, and supported development of national AEFI surveillance system guidelines (*4*).

Much work is still needed to strengthen global vaccine safety monitoring, particularly in WPR, AFR, EUR, and EMR. Barriers to routine reporting of AEFI include 1) lack of reporting tools, 2) poor health care worker understanding of AEFI, 3) weak or poorly coordinated NRA and EPI reporting systems, and 4) health care worker fear of punishment (*6*). Vaccine safety systems are further challenged by a lack of investigative and causality assessment capacity. These issues are particularly relevant in low- and middle-income countries (*7*). Countries that perform consistently well over time have demonstrated a national commitment to addressing these barriers and allocating resources. In Eritrea (in AFR), for example, the number of AEFI reports from EPI increased approximately eightyfold, from 11 in 2016 to 966 in 2018, after NRAs and EPI began an integrated approach to AEFI surveillance. Aided by a GAVI grant,^¶^ Eritrea has conducted vaccine pharmacovigilance, provided AEFI training to health care professionals, and established an AEFI causality assessment committee.

The WHO Global Vaccine Safety Blueprint emphasizes the importance of sharing global vaccine safety data in a national vaccine pharmacovigilance plan (*4*). Sharing data at global and regional levels is critical for monitoring very rare adverse events and for sharing information across countries. At the national level, coordination between EPI and NRA systems is critical to ensure prompt recognition of and response to AEFI, and to protect public health and maintain community trust in the immunization program. Over the past decade, WHO and UMC have collaborated to improve AEFI reporting from country EPIs and NRAs through adaptations in VigiBase-related tools and JRF reporting processes (*8*). Still, only 23.7% of countries reporting to JRF use data that have a combined EPI and NRA data source. The numbers of AEFI reported by NRAs (to UMC) and EPI (to JRF) differ, especially in low- and middle-income countries. Barriers to sharing data at national and global levels include the licensing and operation of AEFI data management and surveillance systems, particularly from data management software developers. To address this, UMC subsidizes country subscription fees for VigiFlow,** an optional national data management system associated with VigiBase.

The findings in this report are subject to at least two limitations. First, the current WHO indicators for minimum safety capacity can provide an inaccurate picture of the quality of the program. For example, many countries only reach minimum capacity for vaccine safety monitoring because they conduct periodic immunization campaigns or other intensified activities, such that an influx in funding, training, and attention to AEFI might contribute to an increase in the number of reports (*9*). Second, the available JRF indicators are unable to capture the performance of AEFI review committees once established, the quality of AEFI investigations, and the quality of surveillance among different target populations. New indicators need to differentiate between serious and nonserious AEFI. Safety Blueprint 2.0, which was endorsed by WHO’s Global Advisory Committee for Vaccine Safety and the Strategic Advisory Group of Experts on Immunization, explains the need for more robust indicators for monitoring safety system performance and stated the case for national, regional, and global investment in safety systems (*10*).

Despite overall progress across the WHO regions in achieving minimum indicators of vaccine safety monitoring, new indicators for monitoring global immunization safety performance are needed to better reflect program functionality. Continued efforts will be vital to address barriers to routine reporting of AEFI, build national capacity for AEFI investigation and data management, and improve sharing of AEFI data at national, regional, and global levels.

**Summary**

**What is already known about this topic?**

Assessing vaccination safety is important to maintaining public confidence in immunization programs. Reporting of adverse events following immunization (AEFI) can be hampered by uncoordinated action between national regulatory authorities and national Expanded Programmes on Immunization.

**What is added by this report?**

During 2010–2019, countries with AEFI review committees increased from 94 (48.5%) to 129 (66.5%) of 194, and those reporting ≥10 AEFI per 100,000 surviving infants increased from 80 (41.2%) to 109 (56.2%). In 2019, however, only 46 (23.7%) reported combined data from national regulatory authorities and Expanded Programmes on Immunization.

**What are the implications for public health practice?**

Updated AEFI surveillance indicators, reduced barriers to reporting, and improved coordination among authorities are needed to strengthen national vaccine safety surveillance systems.

## Footnotes

* Defined as “information (from one or multiple sources) which suggests a new potentially causal association, or a new aspect of a known association, between an intervention and an event or set of related events, either adverse or beneficial, that is judged to be of sufficient likelihood to justify verificatory action.”

† Belarus, Canada, Democratic Republic of the Congo, Guatemala, Haiti, Indonesia, Japan, Monaco, Morocco, Peru, Philippines, Russian Federation, San Marino, Slovakia, Somalia, Timor-Leste, and the United States.

§ Serious AEFI are those that are life-threatening, result in hospitalization or a prolongation of hospitalization, result in persistent or substantial disability, or where the outcome is a birth defect or death, as defined by WHO. Nonserious AEFI are those that do not pose a potential risk to the health of the recipient.

¶
https://www.gavi.org

**
https://www.who-umc.org/global-pharmacovigilance/vigiflow/
