# *Erratum:* Vol. 70, No. 15

**DOI:** 10.15585/mmwr.mm7031a2

**Published:** 2021-08-06

In the report, “Progress in Immunization Safety Monitoring — Worldwide, 2010–2019,” in the figure on p. 548 (Figure 1), six countries were depicted on the map as having data sources “not available,” but should have been depicted as having the following data sources: Djibouti, Expanded Programmes on Immunization (EPI); Ecuador, national regulatory authorities (NRA); Guinea-Bissau, EPI; Mauritius, EPI; Saint Vincent and the Grenadines, EPI; and Seychelles, EPI. The corrected figure is below.

**FIGURE 1:**
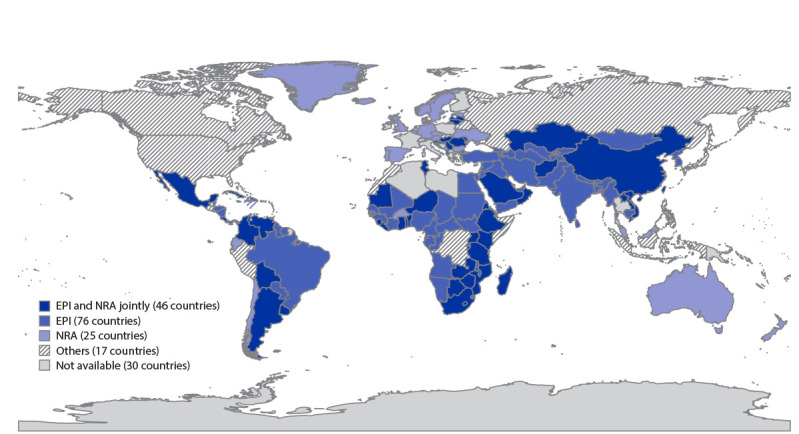
Sources of data for adverse events following immunization reported on the WHO/UNICEF Joint Reporting Form — worldwide, 2019 **Abbreviations:** EPI = Expanded Programmes on Immunization; NRA = national regulatory authorities; WHO = World Health Organization.

